# Excess Neonatal Testosterone Causes Male-Specific Social and Fear Memory Deficits in Wild-Type Mice

**DOI:** 10.1523/ENEURO.0020-25.2025

**Published:** 2025-06-27

**Authors:** Pravda Quiñones-Labernik, Kelsey L. Blocklinger, Matthew R. Bruce, Emily Hagan, Danielle Preuschl, Charlotte Tesar, Sarah L. Ferri

**Affiliations:** ^1^Department of Neuroscience and Pharmacology, University of Iowa, Iowa City, Iowa 52242; ^2^Carver College of Medicine, University of Iowa, Iowa City, Iowa 52242; ^3^Temple University, Philadelphia, Pennsylvania 19122; ^4^Department of Pediatrics, Iowa Neuroscience Institute, University of Iowa, Iowa City, Iowa 52242

**Keywords:** fear conditioning, neurodevelopment, sex differences, social behavior, testosterone

## Abstract

Neurodevelopmental disorders disproportionately affect males compared with females. The biological mechanisms of this male susceptibility or female protection have not been identified. There is evidence that fetal/neonatal gonadal hormones, which play a pivotal role in many aspects of development, may contribute. Here, we investigate the effects of excess testosterone (T) during a critical period of sex-specific brain organization on social approach and fear learning behaviors in C57BL/6J wild-type mice. Male, but not female, mice treated with T on the day of birth (Postnatal Day 0; PN0) exhibited decreased social approach as juveniles and decreased contextual fear memory as adults, compared with vehicle (veh)-treated controls. These deficits were not driven by anxiety-like behavior or changes in locomotion or body weight. Mice treated with the same dose of T on PN18, which is outside of the critical period of brain masculinization, did not demonstrate impairments compared with the veh group. These findings indicate that excess T during a critical period of early development, but not shortly after, induces long-term deficits relevant to the male sex bias in neurodevelopmental disorders.

## Significance Statement

Excess testosterone (T) during a critical period of sex-specific brain organization results in male-specific social and cognitive deficits in mice, while T treatment outside of this developmental window did not alter behavior. This time-sensitive, brief hormonal dysregulation induces long-term changes and may be involved in the male sex bias in neurodevelopmental disorders.

## Introduction

Neurodevelopmental and neuropsychiatric conditions are prevalent and can be difficult to treat; they are emotionally, physically, and financially taxing to affected individuals, their families, and communities (Zablotsky et al., 2019). Therefore, it is crucial to determine developmental processes impacting vulnerability to these disorders, many of which affect males and females divergently. For example, autism spectrum disorder (ASD) and attention-deficit/hyperactivity disorder (ADHD) affect males at a ∼4 and ∼2 to 1 ratio, respectively (Davies, 2014; Yang et al., 2022; Maenner et al., 2023). Age of onset, severity, presentation of symptoms, and response to treatment also differ in males and females. The mechanisms underlying these sex differences are not fully understood, but elucidating them would significantly improve risk assessment, early intervention, and targeted treatments.

One of the earliest developmental processes likely involved in sex-sensitive susceptibility or sex differences in presentation is gonadal hormone exposure during prenatal and neonatal periods. Human male testosterone (T) production surges mid-gestation and shortly after birth, while male rodents have highest levels several days before and after birth. Females of those species are exposed to significantly lower levels of gonadal hormones during early development (Gillies and McArthur, 2010; McCarthy, 2011). It is during this critical period that sex differences in the brain are permanently organized, and during which the brain may be particularly sensitive to hormonal dysregulation, leading to sex-specific vulnerabilities to neurodevelopmental deficits. In rodents, sex hormone-mediated effects on brain development are complete by Postnatal Day (PN)10 (Davis et al., 1996; McCarthy et al., 2018).

Several studies have correlated indirect measures of fetal T exposure such as digit ratio (2D:4D) or facial landmark masculinity or levels of T or steroidogenic factors in amniotic fluid, maternal blood, and cord blood, with ASD-relevant traits or increased likelihood of neurodevelopmental disorder diagnosis (Manning et al., 2001; Lutchmaya et al., 2002a,b; Chapman et al., 2006; De Bruin et al., 2006; Knickmeyer and Baron-Cohen, 2006; Milne et al., 2006; Auyeung et al., 2010, 2012; Baron-Cohen et al., 2015; Park et al., 2017; McKenna et al., 2021; Firestein et al., 2022). There are also a number of conditions in which a fetus may be exposed to excess T derived from the mother or placenta that are associated with increased risk of neurodevelopmental disorder diagnosis in offspring, including congenital adrenal hyperplasia, polycystic ovarian syndrome (PCOS), pre-eclampsia, maternal diabetes, and maternal stress (Lai et al., 2011; Codner et al., 2012; Kumar et al., 2018; Xiang et al., 2018; Gumusoglu et al., 2020; Rowland and Wilson, 2021; Li et al., 2023; Meng et al., 2023).

The role of fetal and neonatal gonadal hormones in hypothalamic structure and function and later reproductive behavior has been a focus of study for some years, but more recently our knowledge of their role in sex differences in other brain regions and behaviors has been expanding. Supporting the broader idea that early sex hormone signaling can shape neurodevelopmental and long-term behavior, animal studies have shown that prenatal exposure to androgens alters neurochemistry and produces sex-specific behavioral outcomes, including those related to reward sensitivity, such as alcohol drinking and amphetamine- and methylphenidate-induced locomotor activity (Dib et al., 2018; Huber et al., 2018; Elgueta-Reyes et al., 2022). Prenatal manipulation of androgen receptor activity in mice has also been shown to produce sex-specific morphological outcomes relevant to those observed in humans, such as digit ratio and body morphology (Huber et al., 2017). Given that many neuropsychiatric conditions present with changes in emotional response and impairments in approach and avoidance behaviors, which can be sex-specific, the present study sought to determine the effects of neonatal T on sociability and fear.

**Figure 1.**
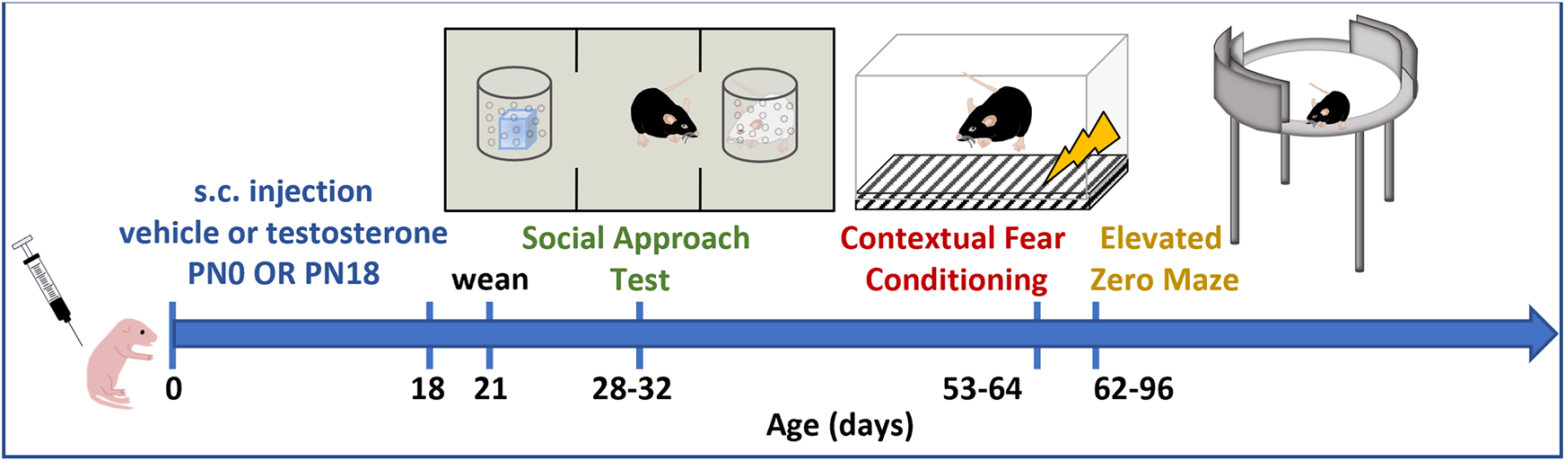
Experimental overview. Pups were injected subcutaneously on the day of birth (PN0) with T or oil veh then remained with the dam and sire until weaning at PN21, after which they were subjected to a weight study (not shown here, PN2–60) or social approach test at PN28–32 and contextual fear conditioning at PN53–64 or EZM at PN62–96. Another group of mice was administered the same dose of T or veh on PN18, weaned at PN21, and underwent social approach test at PN28–32 and contextual fear conditioning at PN53–64. Extended Data Figure 1-1 shows numbers of individual animals and litters used for each experiment.

Social behavior is disrupted in ASD, ADHD, schizophrenia, PTSD, psychopathy, and others (Nijmeijer et al., 2008; Kennedy and Adolphs, 2012; Homberg et al., 2016; Frye, 2018; Porcelli et al., 2019; Scoglio et al., 2022). Many social behaviors exhibit sex differences and are sensitive to gonadal hormones and endocrine disruptors (Bell, 2018). Acquisition of conditioned fear and fear extinction is impaired in psychopathy, PTSD, anxiety, and other disorders (Veit et al., 2013; VanElzakker et al., 2014). The ability to interpret and transmit both social and fear cues are critically important for physical and psychological well-being across many species. Here, we aimed to determine the effects of brief steroid hormone dysregulation during a critical developmental timepoint on social approach and fear memory behaviors. Rodents are a valuable experimental model in which manipulation is well controlled in timing, consistent, and causal. We administered a single dose of T that has been shown to masculinize a female rodent brain to male and female C57BL/6J pups on the day of birth, during the critical period of sex-specific brain organization (Davis et al., 1996; Goel and Bale, 2008; Hisasue et al., 2010; Seney et al., 2012; Ghahramani et al., 2014; McCarthy et al., 2018). We tested the mice in the three-chamber social approach assay as juveniles and in contextual fear conditioning as adults (Fig. 1). We found that a single administration of T during a critical period of brain development (PN0), but not after (PN18), resulted in male-specific deficits in social approach and contextual fear conditioning but no changes in body weight or anxiety-like behavior compared with males treated with vehicle (veh).

10.1523/ENEURO.0020-25.2025.f1-1 Figure 1-1 Numbers of individual animals and litters used for each experiment. Download Figure 1-1, TIF file.

## Materials and Methods

### Animals

Experiments were conducted in accordance with the University of Iowa Institutional Animal Care and Use Committee (IACUC) policies and approved protocols and according to ARRIVE guidelines. Mice were maintained consistent with the Guide for the Care and Use of Laboratory Animals. Mice were housed in a temperature- and humidity-controlled environment (22°C and 55 ± 5%, respectively). All animals were housed on a 12 h light/dark cycle (lights on at 9:00 A.M.). Food and water were available *ad libitum*. C57BL/6J (B6) mice were obtained from The Jackson Laboratory (#000664) to establish breeding cages, which contained one dam and one sire, which remained with the pups until they were weaned. Litters were randomly divided into two experimental conditions: treatment with T or veh (see below), receiving treatment on the day of birth (PN0) or on PN18. On PN21 mice were weaned and housed in groups of 2–5 same-sex littermates per cage, unless otherwise specified. Gonadectomized A/J mice (#000646) were obtained from The Jackson Laboratory and were used as a social stimulus in the social behavioral assay. A total of 234 mice were used for behavioral testing; 21 were excluded due to video corruption (3), inactivity during the testing period (2), or statistical outliers (>2SD from the mean; 16). Of mice treated on PN0, one cohort of 2–3 litters/group was used in weight studies, another (3–5 litters/group) to test anxiety-like behavior, and another in the social approach test (5–8 litters), with a subset undergoing contextual fear conditioning (4–5 litters), further outlined below. Three to four litters of mice treated on PN18 completed social approach and fear memory tests at PN28–32 and 53–64, respectively. Seven of sixty mice treated at PN0 and 7/51 treated at PN18 underwent only fear conditioning as adults due to equipment failure. Litters represented in each experiment are shown in Extended Data Figure 1-1.

### T treatment

T propionate (Sigma-Aldrich; 100 µg in 20 µl sesame oil, a dose previously shown to induce brain masculinization in a female rodent; Goel and Bale, 2008; Hisasue et al., 2010; Seney et al., 2012; Ghahramani et al., 2014; Nakachi et al., 2015) or veh (20 µl sesame oil) was administered subcutaneously at one of two timepoints: to pups on the day of birth (PN0) or on PN18. Each individual mouse within a litter received the same treatment, so experimental cages of littermates were all of the same treatment group. Because each mouse pup was injected individually and dams were left unperturbed, each data point represents a single animal, but litter effects were explored in Extended Data Figures.

Breeding cages were checked 2–4 times per day for litters, and pups were injected usually within 4, but <12, hours of birth. Neonates were separated from the dam for <2 min and gently scruffed for the subcutaneous injection. Following treatment, pups were returned to their homecage with the dam and sire until weaning.

### Weight measurement

In an independent cohort of animals, to avoid repeated handling prior to behavioral assays, weights were measured every 2 d, beginning on Day 2 after birth (PN2), until Day 12. Then, weights were recorded on Days 21, 30, and 60 to monitor treatment effects.

### Social approach test

The social approach test was conducted in PN28–32 mice, in a dimly lit room (<5 lux), using a black Plexiglass arena (10 × 20.5 × 9 in) that had three chambers devoid of top and bottom, which was placed on a clear Plexiglass table over a clean absorbent pad. Identical bottomless and topless clear cylinders were placed at the center of both outer arena chambers. Each clear cylinder featured one end with small breathing holes which facilitated air circulation and enabled visual and olfactory exploration. Flat lids were placed on top of the cylinders and secured with small paperweights. The arena was illuminated from below using infrared light. Testing sessions were recorded from an overheard-positioned camera (Basler Ace GIGE). The behavioral assay consisted of two 10 min phases: a “Habituation” phase followed by a “Choice” phase. During the habituation phase, the test mouse could *ad libitum* explore the chamber and empty cylinders. Following the completion of the habituation phase, a novel object (Duplo block) was introduced into one cylinder, while a novel social stimulus, a same-sex gonadectomized A/J mouse, was placed in the opposite cylinder. Again, the mouse was able to *ad libitum* explore for 10 min during the choice phase. Distance traveled and duration of sniffing of each cylinder were quantified using the Noldus EthoVision XT video tracking software. A preference index (PI) was calculated for each phase: (time spent sniffing social cylinder (empty in habituation or containing novel mouse during choice phase) − time spent sniffing nonsocial cylinder (empty or novel object)) / (total sniffing time). A PI of 0 indicates no preference for either cylinder (equal sniffing of each) and PI of 1 indicates 100% sniffing of social cylinder in the choice phase (or empty cylinder in the habituation phase).

### Contextual fear conditioning

After initial data collection in the social approach test, we decided to test contextual fear conditioning in subsequent mice. Of the 69 animals treated at PN0 used in the social approach test at PN28–32, 44 underwent contextual fear conditioning at PN53–64 to avoid testing during the pubertal period. Of the mice treated at PN18, 44 were tested at PN28–32 for social approach and all were subsequently tested at PN53–64 for fear conditioning plus an additional 8 that did not undergo social testing due to a scheduling issue. Mice were singly housed 4–7 d prior to conditioning and handled 2–3 min each for 3 consecutive days prior to the assay. In our hands, this brief single-housing and handling protocol facilitates learning and avoids cagemate fighting, which we often observe in group-housed mice post-shock. On the day of training, each mouse was placed inside a chamber with electrified metal grid flooring (CleverSys) inside a sound-attenuating box (Med Associates) for a duration of 3 min. During the initial 2 min and 28 s, the mice were allowed to *ad libitum* explore the chamber, which served as a “baseline” period. After this time, a single 1.5 mA footshock was delivered to the mice for 2 s. The mice were removed 30 s following the shock. The test session was conducted 24 h later, during which the mice were placed in the same chamber for a period of 5 min. The Cleversys Freezescan software was utilized to record the freezing behavior of the mice.

### Elevated zero maze

A separate cohort of naive adult male and female mice (PN62–96) was utilized to assess anxiety-like behavior in PN0 T- and veh-treated mice using an elevated zero maze (EZM). The EZM is an elevated (19.75 in) ring-shaped runway with two open arms and two opposing closed arms (outer diameter, 24 in; inner diameter, 20 in). The open arms are devoid of walls resulting in an exposed environment, while the closed arms (6 in high) are enclosed with walls. The EZM was positioned beneath a camera (Basler Ace GIGE) in 250 lux lighting conditions. The MediaRecorder software was used to record the trials. Mice were placed on a boundary between an open and closed area facing the closed area. Each mouse was given a 5 min trial, during which they were allowed to *ad libitum* roam the maze. The experimenter positioned themselves behind a white curtain throughout the trial. The maze was cleaned with paper towels and 70% ethanol between each trial. The Noldus EthoVision XT video tracking software was used to analyze the time spent in the open versus closed areas and total distance traveled within the arena.

### Statistical analysis

Statistical analysis was performed in GraphPad Prism 9. Weight data were analyzed using a repeated-measure (RM) three–way ANOVA to determine main effects of time, sex, treatment, and interactions. For all other data, a two-way ANOVA was performed with sex and treatment as main effects, and a sex × treatment interaction was also tested. A Tukey post hoc test was used when appropriate. Spearman's test was used to determine heteroscedasticity and Shapiro–Wilk for test of normality. Log transformation was used in case of violations; however, raw data are shown in the graph for clarity. Significance was set to *p* < 0.05. Bar graphs and error bars represent mean ± SEM, and individual data points are shown. *η*^2^ values were used for effect size estimations.

## Results

### A single T treatment on the day of birth results in male-specific social approach deficits in juveniles

**Figure 2.**
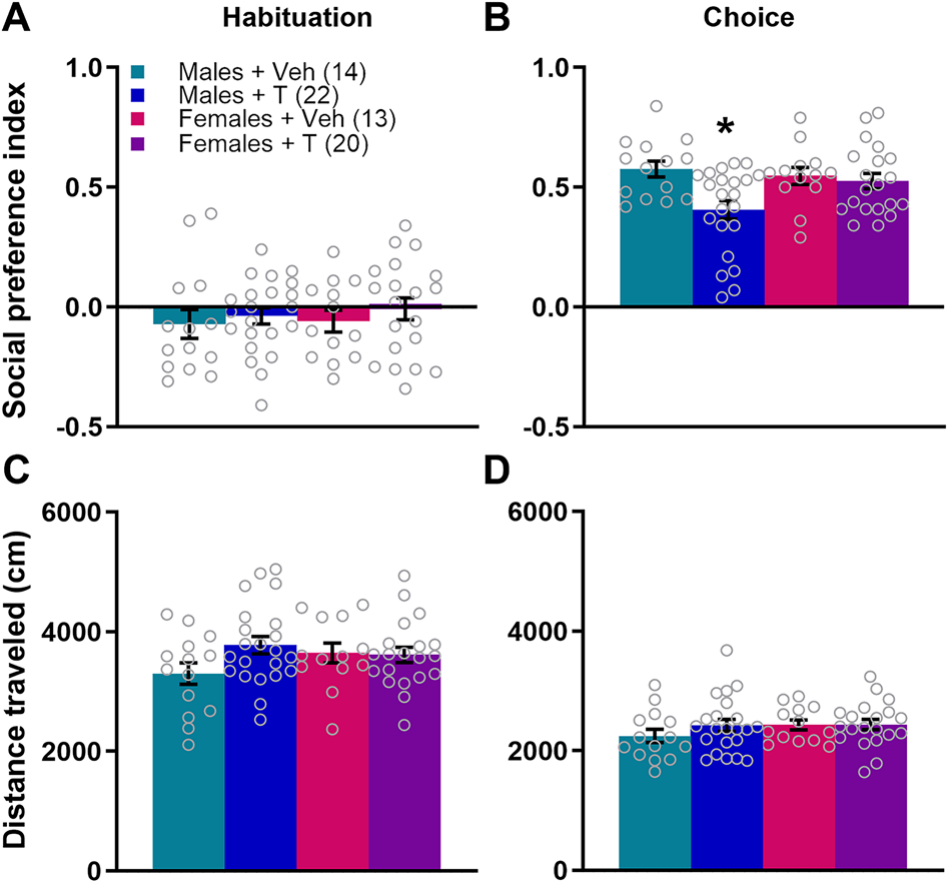
T administration on the day of birth induces social approach deficits in adolescent males. ***A***, During a 10 min habituation period of the social approach test, all experimental groups had a similar PI. ***B***, Males treated neonatally with T had a significantly lower social PI than males or females treated with veh or females treated with T during the choice phase of the social approach test. Data were log transformed for statistical analysis but the graph depicts the raw data. There were no differences across groups in distance traveled during the social approach test in either the habituation ***C*** or choice phase ***D***. *p < 0.05. Error bars indicate mean ± SEM. Data points represent individual mice, but see Extended Data Figure 2-1 for litter averages.

We first aimed to determine whether excess neonatal T would affect social approach behavior. For the PI in the habituation phase, a two-way ANOVA revealed no main effect of sex (*F*_(1,65)_ = 0.200; *p* = 0.656; *η*^2^ = 0.003), treatment (*F*_(1,65)_ = 8.17; *p* = 0.370; *η*^2^ = 0.012), or sex × treatment interaction (*F*_(1,65)_ = 0.034; *p* = 0.855; *η*^2^ = 0.0005; Fig. 2*A*). Social PI data in the choice phase failed Spearman's test, indicating heteroscedasticity, and was subsequently log transformed. A two-way ANOVA of the transformed PI in the choice phase uncovered no main effect of sex (*F*_(1,65)_ = 2.188; *p* = 0.144; *η*^2^ = 0.028), a main effect of treatment (*F*_(1,65)_ = 5.743; *p* = 0.019; *η*^2^ = 0.073), and a statistical trend of a sex × treatment interaction (*F*_(1,65)_ = 3.827; *p* = 0.055; *η*^2^ = 0.049). A Tukey post hoc test indicated that males treated on PN0 with T (Males + T) had significantly lower PI than Males + Veh, Females + Veh, and Females + T (*p* = 0.013, 0.039, and 0.038, respectively; Fig. 2*B*).

10.1523/ENEURO.0020-25.2025.f2-1 Figure 2-1 Testosterone administration on the day of birth induces social approach deficits in adolescent males. (A) During a 10 min habituation period of the social approach test, all experimental groups had a similar preference index (PI). A two-way ANOVA uncovered no significant main effect of sex (F_(1, 22)_=1.542, p=0.227, ƞ^2^ = 0.005) or treatment (F_(1, 22)_ = 0.013, p = 0.909, ƞ^2^ = 0.0005), and no significant interaction (F_(1, 22)_ = 0.125, p = 0.728, ƞ^2^ = 0.005). (B) Males treated neonatally with testosterone had a significantly lower social preference index than males or females treated with vehicle during the choice phase of the social approach test. A two-way ANOVA uncovered a significant main effect of sex (F_(1, 22)_ = 11.130, p = 0.003, ƞ^2^ = 0.306) no main effect of treatment (F_(1, 22)_ = 0.0762, p = 0.392, ƞ^2^ = 0.021), and no significant interaction (F_(1, 22)_ = 1.779, p = 0.196, ƞ^2^ = 0.049). A Tukey post hoc test indicated that males treated on PN0 with testosterone (Males  +  T) had significantly lower freezing than Males  +  Veh and Females  +  Veh, p = 0.016 and 0.033, respectively. (C) There were no differences across groups in distance traveled during the social approach test in the habituation phase. A two-way ANOVA uncovered no significant main effect of sex (F_(1, 22)_ = 0.009, p = 0.925, ƞ^2^ = 0.0003) or treatment (F_(1, 22)_ = 1.521, p = 0.231, ƞ^2^ = 0.064), and no sex x treatment interaction (F_(1, 22)_ = 0.107, p = 0.747, ƞ^2^ = 0.005). (D) There were no differences across groups in distance traveled during the social approach test in the choice phase. A two-way ANOVA uncovered no significant main effect of sex (F_(1, 22)_ = 0.247, p = 0.624, ƞ^2^ = 0.011) or treatment (F_(1, 22)_ = 0.217, p = 0.646, ƞ^2^ = 0.010), and no sex x treatment interaction (F_(1, 22)_ = 0.028, p = 0.870, ƞ^2^ = 0.001). *p<0.05. Bars indicate mean ± SEM. Data points represent litter averages. Download Figure 2-1, TIF file.

Neonatal T treatment had no effects on distance traveled during the social approach test. In juvenile male mice (PN28–32) treated with T at PN0, a two-way ANOVA revealed no main effect of sex (*F*_(1,65)_ = 0.355; *p* = 0.553; *η*^2^ = 0.005) or treatment (*F*_(1,65)_ = 2.008; *p* = 0.161; *η*^2^ = 0.029) and no significant interaction during the habituation phase (*F*_(1,65)_ = 2.655; *p* = 0.108; *η*^2^ = 0.038; Fig. 2*C*). Similarly, during the choice phase, there was no main effect of sex (*F*_(1,65)_ = 1.029; *p* = 0.314; *η*^2^ = 0.015) or treatment (*F*_(1,65)_ = 0.823; *p* = 0.368; *η*^2^ = 0.012) and no significant sex × treatment interaction (*F*_(1,65)_ = 0.697; *p* = 0.407; *η*^2^ = 0.010; two-way ANOVA; Fig. 2*D*). Therefore, the decrease in social approach in juvenile males treated neonatally with T was not an effect of altered locomotor behavior. Litter-averaged social behavior is shown in Extended Data Figure 2-1.

### A single T treatment on the day of birth results in male-specific contextual fear conditioning deficits in adults

**Figure 3.**
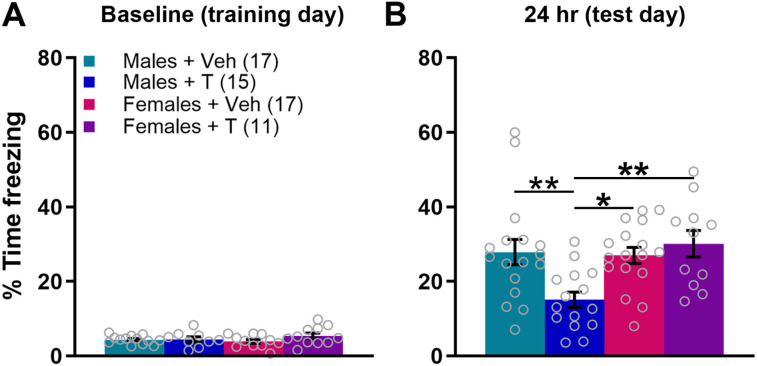
T administration on the day of birth induces fear memory deficits in adult males. ***A***, Baseline freezing prior to contextual fear conditioning was similar regardless of sex or treatment. Extended Data Figure 3-1 shows freezing behavior for the 30 s following the shock. ***B***, Adult males treated on the day of birth exhibited significantly less freezing during the 24 h memory test than those treated with veh or females treated with either veh or T. *p < 0.05; **p < 0.01. Error bars indicate mean ± SEM. Data points represent individual mice, but see Extended Data Figure 3-2 for litter averages.

After initial data collection revealed a male-specific social deficit, we used subsequent animals for contextual fear conditioning following social testing, but we wanted to avoid testing them during puberty, which involves many dynamic changes, so we waited until young adulthood. Therefore, a subset of animals treated with veh or T on PN0 were tested for social approach as juveniles (PN28–32) and then additionally underwent 24 h fear memory testing as adults (PN53–64). The percentage of time spent freezing during the preshock baseline period was similar across groups; a two-way ANOVA showed no main effect of sex (*F*_(1,56)_ = 0.332; *p* = 0.567; *η*^2^ = 0.006), a trend for main effect of treatment (*F*_(1,56)_ = 3.789; *p* = 0.057; *η*^2^ = 0.063), and no significant sex × treatment interaction (*F*_(1,56)_ = 0.556; *p* = 0.459; *η*^2^ = 0.009; Fig. 3*A*). Males treated with T spent significantly less time freezing than males treated on PN0 with veh in the 30 s immediately following the shock (Extended Data Fig. 3-1). A two-way ANOVA of freezing during the 24 h memory test uncovered a significant main effect of sex (*F*_(1,56)_ = 6.084; *p* = 0.017; *η*^2^ = 0.084), a trend toward a main effect of treatment (*F*_(1,56)_ = 2.828; *p* = 0.098; *η*^2^ = 0.039), and a significant interaction (*F*_(1,56)_ = 7.780; *p* = 0.007; *η*^2^ = 0.107). A Tukey post hoc test indicated that males treated on PN0 with T (Males + T) had significantly lower freezing than Males + Veh, Females + Veh, and Females + T (*p* = 0.009, 0.017, and 0.005, respectively; Fig. 3*B*). Extended Data Figure 3-2 shows litter data.

10.1523/ENEURO.0020-25.2025.f3-1 Figure 3-1 Adult males treated with testosterone on PN0 exhibited significantly less freezing during the 30 s following a single footshock on the training day of contextual fear conditioning. A two-way ANOVA uncovered trends towards significance for main effects of both sex (F_(1, 55)_ = 3.034, p = 0.087, ƞ^2^ = 0.047) and treatment (F_(1, 55)_ = 2.961, p = 0.091, ƞ^2^ = 0.045), and a significant interaction (F_(1, 55)_ = 4.280, p = 0.043, ƞ^2^ = 0.066). A Tukey post hoc test indicated that males treated on PN0 with testosterone (Males  +  T) had significantly lower freezing than Males  +  Veh (p = 0.036) and a trend towards significantly less freezing than Females  +  Veh, p = 0.065 and Females  +  T, p = 0.071. *p<0.05, #p<0.10. Bars indicate mean ± SEM. Data points represent individual mice. Download Figure 3-1, TIF file.

10.1523/ENEURO.0020-25.2025.f3-2 Figure 3-2 Testosterone administration on the day of birth induces fear memory deficits in adult males. (A) Baseline freezing prior to contextual fear conditioning was similar regardless of sex or treatment. A two-way ANOVA uncovered no significant main effect of sex (F_(1, 14)_ = 0.449, p = 0.514, ƞ^2^ = 0.025) or treatment (F_(1, 14)_ = 2.887, p = 0.111, ƞ^2^ = 0.162), and no sex x treatment interaction (F_(1, 14)_ = 0.389, p = 0.543, ƞ^2^ = 0.022). (B) Adult males treated with T on PN0 exhibited significantly less freezing during the 24 hr memory test than those treated with veh or females treated with T. A two-way ANOVA uncovered a trend toward a main effect of sex (F_(1, 14)_ = 3.985, p = 0.066, ƞ^2^ = 0.142) and treatment (F_(1, 14)_ = 3.257, p = 0.093, ƞ^2^ = 0.116), and a significant interaction (F_(1, 14)_ = 5.609, p = 0.033, ƞ^2^ = 0.200). A Tukey post hoc test indicated that Males  +  T had significantly lower freezing than Males  +  Veh (p = 0.046) and Females  +  T (p = 0.025), and a trend towards significantly less freezing than Females  +  Veh (p = 0.074). *p<0.05, #p<0.10. Bars indicate mean ± SEM. Data points represent litter averages. Download Figure 3-2, TIF file.

### Social and fear memory deficits are not due to differences in body weight or increased anxiety-like behavior

**Figure 4.**
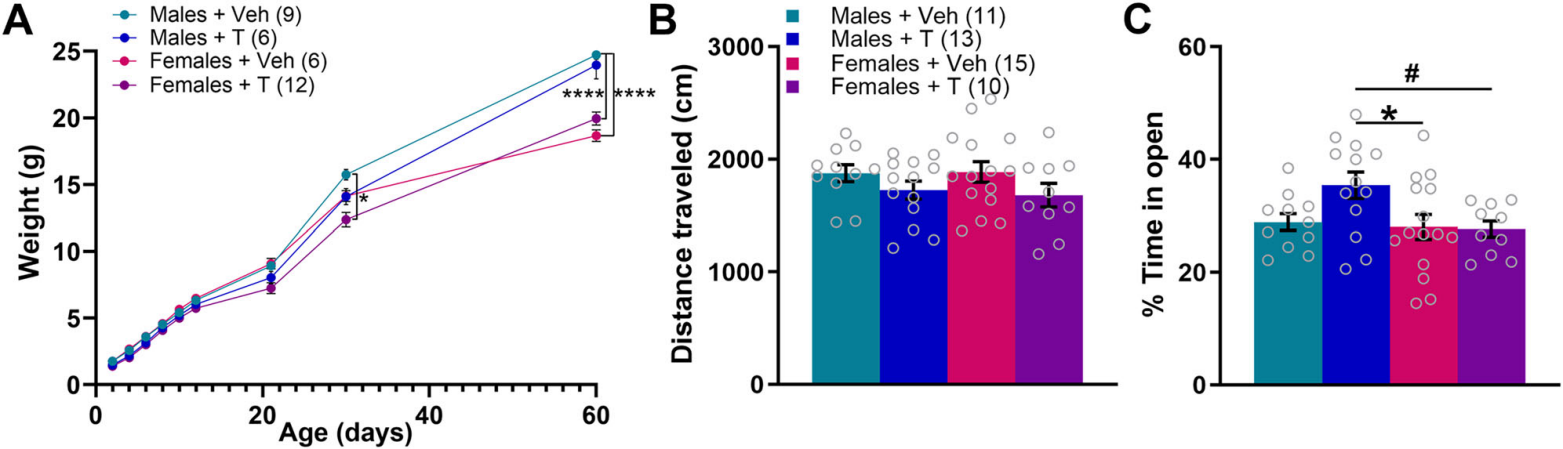
Excess neonatal T-induced effects are not due to changes in weight or anxiety-like behavior. ***A***, T administration on the day of birth does not affect body weight. Mice treated at PN0 with T or veh were weighed on PN2, 4, 6, 8, 10, 12, 21, 30, and 60. Most differences in weight were driven by sex. Specifically, at PN30, males treated with veh weighed significantly more than females treated with T, and at PN60, males treated neonatally with veh weighed significantly more than females treated either with veh or T on the day of birth. ***B***, Naive adult males and females treated neonatally with T traveled similar distances in the EZM as those treated with veh. ***C***, T treatment on the day of birth increased the percentage of time spent in the open arms of the EZM compared with females treated with veh. ^#^*p* < 0.10; *p < 0.05; ****p < 0.0001. Data points represent individual mice, but see Extended Data Figure 4-1 for litter averages.

We then used separate groups of animals to determine whether changes in body weight or anxiety may contribute, indicating a general disruption in growth or development that may affect behavior or movement, or decreased overall exploration or increased avoidance. Mice that underwent treatment with neonatal T had similar body weight to those treated with veh on the day of birth (Fig. 4*A*). A RM three-way ANOVA revealed main effects of age (*F*_(2,60)_ = 2525; *p* < 0.0001; *η*^2^ = 0.886), sex (*F*_(1,29)_ = 12.69; *p* = 0.001; *η*^2^ = 0.004), and treatment (veh vs T; *F*_(1,29)_ = 7.345; *p* = 0.011; *η*^2^ = 0.002) and the following significant interactions: age × sex (*F*_(8,232)_ = 41.04; *p* < 0.0001; *η*^2^ = 0.014), age × treatment (*F*_(8,232)_ = 4.967; *p* < 0.0001; *η*^2^ = 0.002), and age × sex × treatment (*F*_(8,232)_ = 2.556; *p* < 0.011; *η*^2^ = 0.0008), but no sex × treatment interaction (*F*_(1,29)_ = 0.027; *p* = 0.871; *η*^2^ = 7.9 × 10^−6^). A Tukey post hoc test indicated that at age PN30, males treated with veh weighed significantly more than females treated with T (*p* = 0.021) and at PN60, Males + Veh weighed significantly more than both Females + Veh and Females + T (*p* < 0.0001 for both). In summary, most significant differences in weight were due to sex as expected, but there were no significant differences driven by T within sexes at any age.

10.1523/ENEURO.0020-25.2025.f4-1 Figure 4-1 Neonatal testosterone treatment does not affect behavior in the elevated zero maze. (A) Naïve adult males and females treated neonatally with testosterone traveled similar distances in the elevated zero maze as those treated with veh. A two-way ANOVA uncovered a trend toward a main effect of sex (F_(1, 11)_ = 3.297, p = 0.097, ƞ^2^ = 0.226) no significant effect of treatment (F_(1, 11)_ = 0.032, p = 0.861, ƞ^2^ = 0.002), and no significant sex x treatment interaction (F_(1, 11)_ = 0.232, p = 0.639, ƞ^2^ = 0.015). (B) Testosterone treatment on the day of birth did not affect percent time spent in the open arms of the EZM. A two-way ANOVA revealed no significant main effects (sex: F_(1, 11)_ = 0.732, p = 0.411, ƞ^2^ = 0.048, treatment: F_(1,11)_ = 2.073, p = 0.178, ƞ^2^ = 0.136), nor an interaction (F_(1, 11)_ = 2.109, p = 0.174, ƞ^2^ = 0.138). Data points represent litter averages. Download Figure 4-1, TIF file.

In the EZM, there was a trend for naive adult mice treated on the day of birth with T to travel less distance overall compared with those treated with veh on the day of birth (Fig. 4*B*). A two-way ANOVA of total distance traveled over the 5 min test revealed no main effect of sex (*F*_(1,45)_ = 0.034; *p* = 0.855; *η*^2^ = 0.000), a trend toward a significant main effect of treatment (*F*_(1,45)_ = 3.914; *p* = 0.054; *η*^2^ = 0.080), and no significant interaction between the two (*F*_(1,45)_ = 0.095; *p* = 0.760; *η*^2^ = 0.002). Males treated with T on PN0 exhibited slightly less anxiety-like behavior as measured by the increased percentage of time spent in open arms in the EZM (Fig. 4*C*). A two-way ANOVA of the percentage of time spent in the open arms revealed a main effect of sex (*F*_(1,45)_ = 4.400; *p* = 0.042; *η*^2^ = 0.069), no main effect of treatment (*F*_(1,45)_ = 2.202; *p* = 0.145; *η*^2^ = 0.013), and a trend toward a significant sex × treatment interaction (*F*_(1,45)_ =2.283; *p* = 0.099; *η*^2^ = 0.044). A Tukey post hoc test indicated that males treated on PN0 with T (Males + T) spent a significantly higher percentage of time in the open arms than Females + Veh (*p* = 0.043) and exhibited a trend of increased percentage of time in open arms compared with Females + T (*p* = 0.059). Results for litters are shown in Extended Data Figure 4-1.

### A single T treatment on PN18 does not induce social approach deficits in juveniles

**Figure 5.**
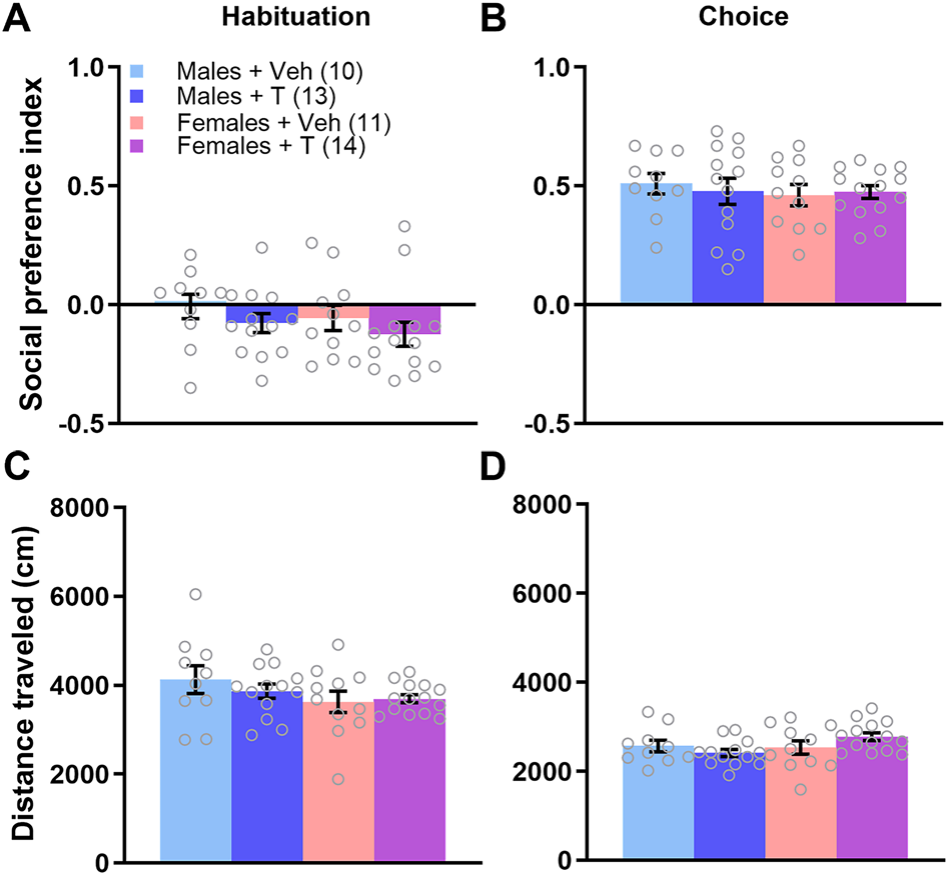
T administered on PN18 does not affect social approach behavior. ***A***, Male and female adolescent mice exhibited no significant differences in PI during habituation regardless of treatment with veh or T on PN18. ***B***, T treatment on PN18 had no effect on PI during the choice phase. ***C***, During habituation, all experimental groups traveled similar distances. ***D***, During the choice phase, all experimental groups traveled similar distances. Data points represent individual mice, but see Extended Data Figure 5-1 for litter averages.

Next, we wanted to determine if the same dose of T treatment given later, outside the reported critical period of brain masculinization (Davis et al., 1996; McCarthy et al., 2018), would cause similar social impairments. For PI during the habituation phase of juvenile mice treated with veh or T on PN18, a two-way ANOVA showed no main effects or interaction (*F*_(1,44)_ = 0.924; *p* = 0.342; *η*^2^ = 0.020 for sex; *F*_(1,44)_ = 1.947; *p* = 0.170; *η*^2^ = 0.042 for treatment; *F*_(1,44)_ = 0.0003; *p* = 0.986; *η*^2^ = 0.000 for interaction; Fig. 5*A*). During the choice phase of the social approach test, all groups had a similar PI; a two-way ANOVA produced no main effects of sex (*F*_(1,44)_ = 0.337; *p* = 0.564; *η*^2^ = 0.008) or treatment (*F*_(1,44)_ = 0.048; *p* = 0.828; *η*^2^ = 0.001) and no interaction of the two (*F*_(1,44)_ = 0.270; *p* = 0.606; *η*^2^ = 0.006; Fig. 5*B*).

10.1523/ENEURO.0020-25.2025.f5-1 Figure 5-1 Testosterone administered on PN18 does not affect social approach behavior in juveniles. (A) Male and female adolescent mice exhibited no significant differences in PI during habituation regardless of treatment with veh or T on PN18. A two-way ANOVA revealed no significant main effects of sex (F_(1, 9)_ = 1.210, p = 0.300, ƞ^2^ = 0.106) or treatment (F_(1, 9)_ = 1.082, p = 0.325, ƞ^2^ = 0.094), and no significant interaction (F_(1, 9)_ = 0.125, p = 0.728, ƞ^2^ = 0.005). (B) Testosterone treatment on PN18 had no effect on PI during the choice phase. A two-way ANOVA uncovered no significant main effects of sex (F_(1, 9)_ = 0.183, p = 0.679, ƞ^2^ = 0.019) or treatment (F_(1, 9)_ = 0.005, p = 0.947, ƞ^2^ = 0.0005), and no significant interaction (F_(1, 9)_ = 0.260, p = 0.622, ƞ^2^ = 0.028). (C) During habituation, all experimental groups traveled similar distances. A two-way ANOVA uncovered no significant main effects of sex (F_(1, 9)_ = 1.828, p = 0.209, ƞ^2^ = 0.165) or treatment (F_(1, 9)_ = 0.001, p = 0.976, ƞ^2^ = 0.000), and no significant interaction (F_(1, 9)_ = 0.361, p = 0.563, ƞ^2^ = 0.033). (D) During the choice phase, all experimental groups traveled similar distances. A two-way ANOVA revealed no significant main effects of sex (F_(1, 9)_ = 0.378, p = 0.554, ƞ^2^ = 0.030) or treatment (F_(1, 9)_ = 0.326, p = 0.582, ƞ^2^ = 0.026), and no significant interaction (F_(1, 9)_ = 2.362, p = 0.159, ƞ^2^ = 0.190). Data points represent litter averages. Download Figure 5-1, TIF file.

For distance traveled in the habituation phase, a two-way ANOVA revealed a trend toward a main effect of sex (*F*_(1,44)_ = 2.831; *p* = 0.100; *η*^2^ = 0.060), no main effect of treatment (*F*_(1,44)_ = 0.230; *p* = 0.634; *η*^2^ = 0.005), or sex × treatment interaction (*F*_(1,44)_ = 0.672; *p* = 0.417; *η*^2^ = 0.014; Fig. 5*C*). A two-way ANOVA of the distance traveled in the choice phase uncovered no significant main effect of sex (*F*_(1,44)_ = 2.217; *p* = 0.144; *η*^2^ = 0.044), no main effect of treatment (*F*_(1,44)_ = 0.133; *p* = 0.717; *η*^2^ = 0.003), and a trend toward a sex × treatment interaction (*F*_(1,44)_ = 3.196; *p* = 0.081; *η*^2^ = 0.063; Fig. 5*D*). Social approach data for litters are shown in Extended Data Figure 5-1.

### A single T treatment on PN18 does not induce contextual fear conditioning deficits in adults

**Figure 6.**
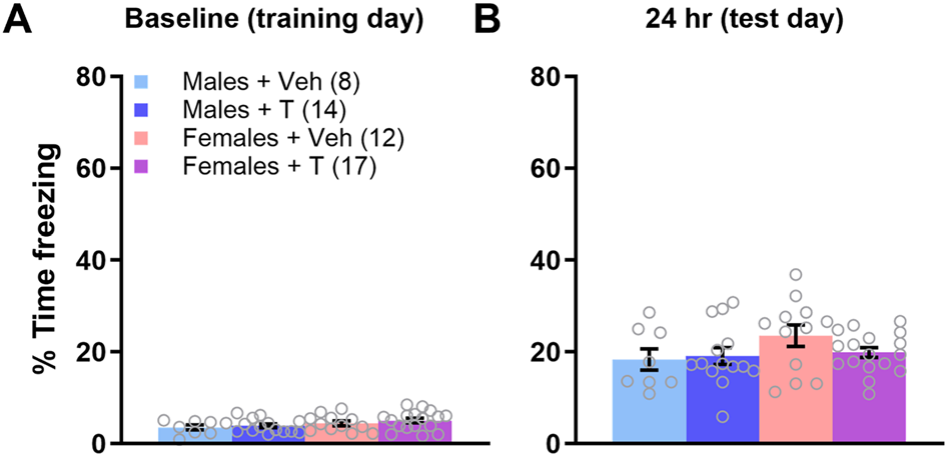
T administration on PN18 does not affect contextual fear conditioning in adults. ***A***, Treatment with T on PN18 did not affect the percentage of time spent freezing during the baseline measure. Extended Data Figure 6-1 shows freezing behavior for the 30 s following the shock. ***B***, There were no group differences in freezing during the 24 h test of contextual fear memory. Error bars indicate mean ± SEM. Data points represent individual mice, but see Extended Data Figure 6-2 for litter averages.

A two-way ANOVA of the percentage of time spent freezing during the baseline period demonstrated a trend toward a main effect of sex (*F*_(1,47)_ = 3.887; *p* = 0.055; *η*^2^ = 0.074), no main effect of treatment (*F*_(1,47)_ = 0.779; *p* = 0.382; *η*^2^ = 0.015), and no significant phase × sex interaction (*F*_(1,47)_ = 0.081; *p* = 0.778; *η*^2^ = 0.002; Fig. 6*A*). For the 30 s following the shock, females treated on PN18 with T spent an increased percentage of time freezing compared with males treated with veh or T (Extended Data Fig. 6-1). Twenty-four hours later, freezing percentages were similar across all groups. A two-way ANOVA showed no significant main effect of sex (*F*_(1,47)_ = 2.513; *p* = 0.120; *η*^2^ = 0.049) or treatment (*F*_(1,47)_ = 0.605; *p* = 0.441; *η*^2^ = 0.012), and no interaction (*F*_(1,47)_ = 1.421; *p* = 0.239; *η*^2^ = 0.028; Fig. 6*B*). Litter-averaged fear conditioning results are in Extended Data Figure 6-2.

10.1523/ENEURO.0020-25.2025.f6-1 Figure 6-1 Testosterone administration on the day of birth causes increased freezing in females in the 30 s following a single footshock on the training day of contextual fear conditioning. A two-way ANOVA uncovered a significant main effect of sex (F_(1, 47)_ = 9.629, p = 0.003, ƞ^2^ = 0.142) but not treatment (F_(1, 47)_ = 0.742, p = 0.394, ƞ^2^ = 0.011), and a significant sex x treatment interaction (F_(1, 47)_ = 5.651, p = 0.022, ƞ^2^ = 0.083). A Tukey post hoc test indicated that females treated on PN0 with testosterone had significantly higher freezing than Males  +  Veh (p = 0.047) and Males  +  T (p = 0.0003), and a trend towards significance compared to Females  +  Veh (p = 0.073). *p<0.05, ***p<0.001, #p<0.10. Bars indicate mean ± SEM. Data points represent individual mice. Download Figure 6-1, TIF file.

10.1523/ENEURO.0020-25.2025.f6-2 Figure 6-2 Testosterone administration on PN18 does not affect contextual fear conditioning in adults. (A) Treatment with T on PN18 did not affect percent of time spent freezing during the baseline measure. A two-way ANOVA uncovered a trend toward a main effect of sex (F_(1, 12)_ = 3.725, p = 0.078, ƞ^2^ = 0.206), no main effect of treatment (F_(1, 12)_ = 1.899, p = 0.193, ƞ^2^ = 0.105), and no significant interaction (F_(1, 12)_ = 0.161, p = 0.695, ƞ^2^ = 0.009). (B) There were no group differences in freezing during the 24 hr test of contextual fear memory. A two-way ANOVA revealed no significance in main effects of sex (F_(1, 12)_ = 3.071, p = 0.105, ƞ^2^ = 0.202) or treatment (F_(1, 12)_ = 0.016, p = 0.901, ƞ^2^ = 0.001), and no sex x treatment interaction (F_(1, 12)_ = 0.540, p = 0.477, ƞ^2^ = 0.036). Bars indicate mean ± SEM. Data points represent litter averages. Download Figure 6-2, TIF file.

## Discussion

Our data show that a single T treatment on the day of birth results in both social deficits in juvenile and fear memory deficits in adult, male wild-type (C57BL/6J) mice but has no effect on females. The same single subcutaneous injection of T propionate (100 µg; Goel and Bale, 2008; Hisasue et al., 2010; Seney et al., 2012; Ghahramani et al., 2014; Nakachi et al., 2015) administered on PN18 has no effect on social or fear memory behavior at PN30 or PN60, respectively. Considering that male rodents exhibit peak levels of T several days before and after birth, while females experience negligible exposure to gonadal hormones during early development, PN18 was chosen as a timepoint outside the endogenous T activity (Davis et al., 1996; McCarthy et al., 2018). Therefore, our results indicate that excess T during development is not universally detrimental, but there is a sensitive period in which excess T can disrupt sex-specific neural circuits and even a brief dysregulation of T levels can induce long-lasting effects. Additionally, these findings indicate that both social approach behavior and fear memory exhibit sex differences in vulnerability in early development, which has significant implications for neurodevelopmental and neuropsychiatric disorders that affect males and females differently in terms of prevalence, presentation, or progression.

Notably, neonatal T treatment did not affect mortality; the litter size was 6.735 ± 0.392 for veh and 5.970 ± 0.388 for T; *p* = 0.1700. Additionally, veh-treated mice exhibited behavior statistically equivalent to historical data of ours with mice that were undisturbed as neonates (no neonatal injection); both social preference indices at ∼PN30 and 24 h fear memory results at ∼PN60 were comparable between mice administered veh and never-injected control mice we have collected over various experiments (Schoch et al., 2017; Ferri et al., 2016, 2020, 2021). The observed deficits were not due to changes in body weight or motor activity that may affect general function or indicate global disruptions in physical development. The deficits were also not attributed to heightened anxiety-like behavior, which can co-occur with social deficits (Allsop et al., 2014; Felix-Ortiz et al., 2016). In fact, the males treated with T on the day of birth spent significantly more time in the open arms than females treated on PN0 with veh or T and nonsignificantly more time in the open arms than the control male group. It will be interesting to further investigate related exploratory and risk-assessment behaviors in these mice.

In our study, we utilized contextual fear conditioning as an assay of hippocampal-dependent learning, which is particularly relevant for modeling cognitive aspects of neurodevelopmental and neuropsychiatric disorders. While our data demonstrate a causal relationship between elevated neonatal T and impaired fear memory in males, future work is needed to determine whether this deficit reflects a broader impairment in learning or is specific to fear-related memory processes. In addition, in the PN0 experiments, T-treated males showed significantly reduced and less variable freezing behavior in the 30 s immediately after footshock on the training day compared with controls (Extended Data Fig. 3*A*). In contrast, PN18T-treated females exhibited increased and more variable freezing despite no differences in behavior in the baseline and 24 h memory periods (Extended Data Fig. 6*A*). It is difficult to interpret these results because it is such a short period of time and control animals consistently show high variability in their response to the shock, exhibiting freezing, darting, or attempts to escape. Future experiments should test freezing at additional timepoints, such as 1 and 4 h, investigate extinction learning in these mice, and determine whether pain sensitivity, risk aversion, or estrous cycle, which may influence both behavior and pain sensitivity, contribute to the differences in behavior immediately following footshock.

A crucial next step is to determine the mechanisms by which excess T during a critical period of brain organization induces male-specific social and fear deficits. Early in development, genes on the Y chromosome orchestrate the production of T by the testis in males. T can then be metabolized into dihydrotestosterone, which binds to androgen receptors, or it can be aromatized to estradiol and bind to estrogen receptors. Both processes are important for distinct components of brain masculinization and defeminization (Gillies and McArthur, 2010; McCarthy, 2011). It will be necessary to determine which pathway or if both pathways are disrupted to cause sex-specific social and fear deficits. Importantly, fetoneonatal estrogen-binding proteins, such as alpha-fetoprotein, bind circulating estrogens and prevent their entry into the developing female brain, thereby protecting against estrogen-driven masculinization during critical organizational periods (Bakker et al., 2006). This protective mechanism may help explain why neonatal T treatment in our study resulted in male-specific social and fear memory deficits, while females remained unaffected. Future studies will be important to test the role of estrogen-binding proteins during the neonatal period in T-induced behavioral deficits. Another critical mechanistic question concerns brain regions that may be dysregulated by excess neonatal T. The medial prefrontal cortex, amygdala, and hippocampus play important roles in both social and fear memory behavior and may be disrupted by excess T early in development (Maren, 2001; Kim and Jung, 2006; Marschner et al., 2008; Jasnow et al., 2013; Bickart et al., 2014; Felix-Ortiz and Tye, 2014; LaLumiere, 2014; Bicks et al., 2015; Kim et al., 2016; Zaki et al., 2022; Kietzman and Gourley, 2023). The ventral tegmental area, nucleus accumbens, and cerebellum have also been implicated in the regulation of social behavior (Gunaydin et al., 2014; Carta et al., 2019; Porcelli et al., 2019; Musardo et al., 2022; Solié et al., 2022). Numerous sex differences in morphology and function in these brain regions have been documented, including in area volume, cell number, size, and structure, and most express androgen and estrogen receptors (Premachandran et al., 2020). Finally, fetal/neonatal T during the critical organizational period of brain development has important effects on a number of downstream processes. Neurotransmitter levels, receptor expression, neuropeptide signaling, neurogenesis, synaptic programming, and cell differentiation, migration, and death are influenced by gonadal hormones during development and may be involved in the social and fear memory deficits (Baron-Cohen et al., 2005, 2011; Schaafsma et al., 2017; Ferri et al., 2018). Investigating these potential mechanisms will provide insight into developmental processes involved in impairments associated with neurodevelopmental and other disorders.

While we used a single, moderate dose of T in this study, it will be important to determine the effects of different amounts of T in future studies. This dose and timing were chosen because it has been shown to masculinize a female rodent brain (Goel and Bale, 2008; Hisasue et al., 2010; Seney et al., 2012; Nakachi et al., 2015), but the effects on the male brain and behavior have not been described. Several studies have used excess T exposure in utero; one found that males, but not females, exhibited increased density, instability, and abnormal morphology in dendritic spines of the frontal cortex, and another showed male-specific decreases in corticosterone response following restraint stress (Hatanaka et al., 2015; Wilson et al., 2020). Another study demonstrated that repeated corticosterone injections in pregnant dams increased brain T levels in male fetuses and led to male-specific changes in *N*-methyl-d-aspartate receptor subunit expression, a pathway implicated in both social behavior and neurodevelopmental disorders (Kalinichenko et al., 2023; Brown et al., 2024). This represents a downstream target that our lab is currently investigating. One study administering 10× the dose used here, administered on PN2, and using RNA from the entire PN6 brain, identified 319 genes that were differentially expressed between veh- and T-treated males, and decreases in estrogen receptor- and androgen receptor-responsive gene elements were found in the flanking regions of a number of those genes. In addition, levels of *Esr2* (estrogen receptor β) and *Cyp19a1* (aromatase, the enzyme that converts T to estradiol) were not statistically different in males treated with veh and males treated with T, but *Esr1* (estrogen receptor α; ERα) was downregulated in T-treated male brains. *Esr1* was not different between veh- and T-treated females, however (Nakachi et al., 2015). ERα-knock-out mice exhibit social deficits, and a single-nucleotide polymorphism was reported to correlate with severity in social interaction deficits in a group of children with autism, although ESR1 is not considered a high confidence risk gene for ASD (Ervin et al., 2015; Doi et al., 2018; Enriquez et al., 2021). Therefore, the possibility of ESR1-mediated mechanism of approach and avoidance deficits will be important to examine in our paradigm.

We demonstrated that neonatal T treatment at PN0 but not PN18 causes behavioral deficits, but future investigation of additional timepoints between PN0 and PN18 will be valuable to help pinpoint periods of vulnerability. Additionally, here we treated the entire litter with veh or T, and those same-treatment mice remained housed together; future studies will address whether mixed-treatment mice housed together exhibit social and fear memory deficits similar to those described here. Relatedly, because each neonate was treated individually, each data point represents a single animal. We also demonstrated similar findings with litter averages representing each data point (Extended Data). However, while the dam was left undisturbed, minimizing likelihood of disruptions in maternal care, and we did not observe any changes in dam behavior toward pups injected with T compared with controls, we cannot yet completely rule out subtle changes in maternal care of mice treated with T that may influence later behavior.

Importantly, complications of pregnancy including PCOS, pre-eclampsia, and gestational diabetes that result in increased risk of neurodevelopmental disorders in the offspring involve a number of complex factors in addition to increased levels of T. Likewise, an interaction of genes or environmental insults and sex hormone levels likely contribute to the development of neuropsychiatric conditions, and there is some evidence of this (Schaafsma and Pfaff, 2014; Young and Pfaff, 2014; Baron-Cohen et al., 2015; Schaafsma et al., 2017; Bordt et al., 2024). However, we have shown that excess T in early development alone is sufficient to induce neurodevelopmental deficits in mice, which paves the way for future studies. Obvious ethical constraints prohibit well-controlled manipulations in humans, and it is not possible to safely study T levels in a human fetus midgestation during the critical period of brain masculinization. Relatedly, the relationship of blood or brain levels of T and more accessible samples like maternal blood levels or those measured during amniocentesis, which is usually only indicated in high-risk pregnancies, is not clear. These studies are valuable but are also complicated by heterogeneity in human subjects, lack of information, small sample size, and findings that fail to replicate. Animal models can help identify possible biomarkers that are modifiable as possible treatment targets, as there are several pharmacological agents in use that modulate steroid hormone levels. In conclusion, while we do not present the manipulation in this study as a model of any specific disorder, we propose that it is a valuable paradigm in which dysregulation of sex-specific and time-sensitive developmental pathways can be used to investigate differential vulnerabilities to behavioral deficits, which may be relevant to a number of neuropsychiatric conditions that express sex differences.

## Synthesis

Reviewing Editor: Nathalie Ginovart, University of Geneva Decisions are customarily a result of the Reviewing Editor and the peer reviewers coming together and discussing their recommendations until a consensus is reached. When revisions are invited, a fact-based synthesis statement explaining their decision and outlining what is needed to prepare a revision will be listed below. The following reviewer(s) agreed to reveal their identity: Christian Müller, Anna Radke.

Both reviewers expressed interest in your manuscript and agreed that it provides new insights into how developmental exposure to testosterone affects learning and social behavior. However, a few points were raised that should be addressed in a revision to improve the clarity of the manuscript and enhance its impact.

Please find the reviewer's comments below:

Reviewer 1:

The manuscript investigates the effects of neonatal and juvenile testosterone exposure on adult contextual fear memory and social approach behaviour in a mouse model. The authors treated male and female mice on postnatal day one and alternatively day 18 and tested them as juveniles and as adults. They report a sex-dependent decrease in social approach in juveniles and decrease in contextual fear memory in adult males. This effect was not observed when treated on day 18. Authors conclude that testosterone exerts its effects on behavioural maturation in a strongly age and sex-dependent way. These findings will further add to our understanding of the important effects testosterone may have during development.

Overall, the study addresses an interesting and timely issue. The experiments are well-planned and performed. Analysis of data is appropriate and results are presented in a clear and easy to follow way. Altogether, there are only minor issues that may be addressed before publication can be considered.

Minor:

Literature discussion on the research theme is not always covering key previous research on testosterone effects on behaviour and morphology. Authors may also consider, e.g. Huber SE et al., (2018) Prenatal testosterone-receptor activation determines adult alcohol and water drinking in a gender-specific way, Addiction Biology, 23(3), 904-920; Huber SE (2017) Prenatal androgen-receptor activity has organizational morphological effects in mice, PLOS One 12(11):e0188752; Kalinichenko LS et al. (2023) Sex-specific effects of different types of prenatal stress on foetal testosterone levels and NMDA expression in mice. Behavioural Brain Research, 439, 114225

References in reference list are occasionally incomplete. The authors may wish to add the missing information.

Reviewer 2:

The manuscript "Excess neonatal testosterone causes male-specific social and fear memory deficits in wild-type mice" is based around a group of experiments involving s.c injections of testosterone in PND0 or PND18 male and female pups. The findings were that male mice injected with testosterone on PND0 showed significantly lower freezing than other groups, and lower preference index during the choice phase. Results from PND18 experiments were mostly null, but males + T did travel less than females + T. The data are clearly presented and interesting. This manuscript will make an important contribution to the literature after a few revisions.

Major revisions:

1. Please mention which results had outliers removed, and if doing so impacted the results.

2. It is stated "experimental cages of littermates were all of the same treatment group." Developmental studies often balance treatments across litters, using no more than one male and one female per litter in an experiment. This ensures that the results are not influenced by litter effects. Please note how many litters are represented in each experimental group. A table outlining the groups and sample sizes for each experiment would be useful.

3. Please note which data sets violated normality.

4. Was shock reactivity and freezing during the training session recorded? If so, please analyze and note if there were any differences in the text.

5. The results could reflect a general learning deficit. Please comment on this possibility in the Discussion.

6. The Fetoneonatal E2 Binding Protein (FEBP) protection hypothesis seems very relevant and the data should be discussed in this context.

7. In general, the introduction and discussion could be more targeted toward the behaviors being studied. The focus begins on ASD and ADHD, but the tests used then seem less relevant.

Minor revisions:

1. Line 88 - include university name

2. Please include the dimensions of the EZM.
