# Supplementary figures and images for: Excess Neonatal Testosterone Causes Male-Specific Social and Fear Memory Deficits in Wild-Type Mice
Source: eNeuro. 2025 Jun 27;12(8):ENEURO.0020-25.2025. doi: 10.1523/ENEURO.0020-25.2025 (PMC12320760; doi:10.1523/ENEURO.0020-25.2025)

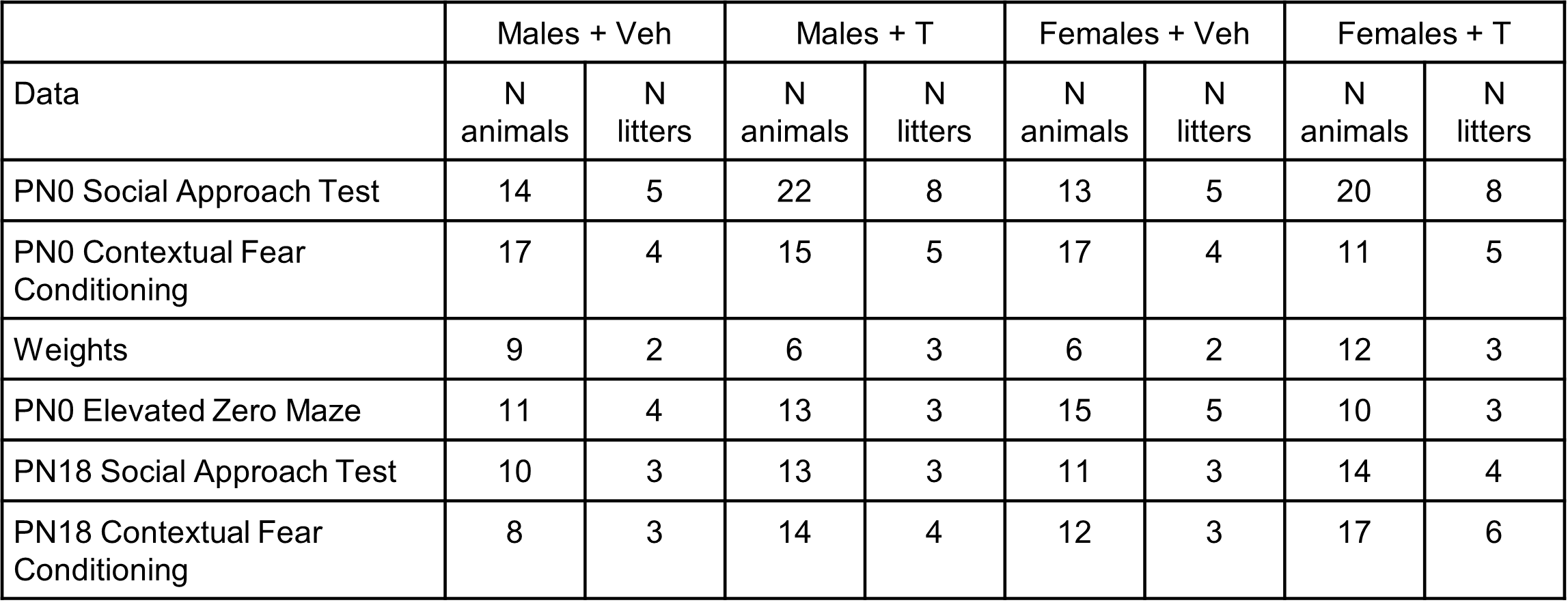

Supplement: Figure 1-1 — Numbers of individual animals and litters used for each experiment. Download Figure 1-1, TIF file. [file eneuro-12-ENEURO.0020-25.2025-s002.tif]

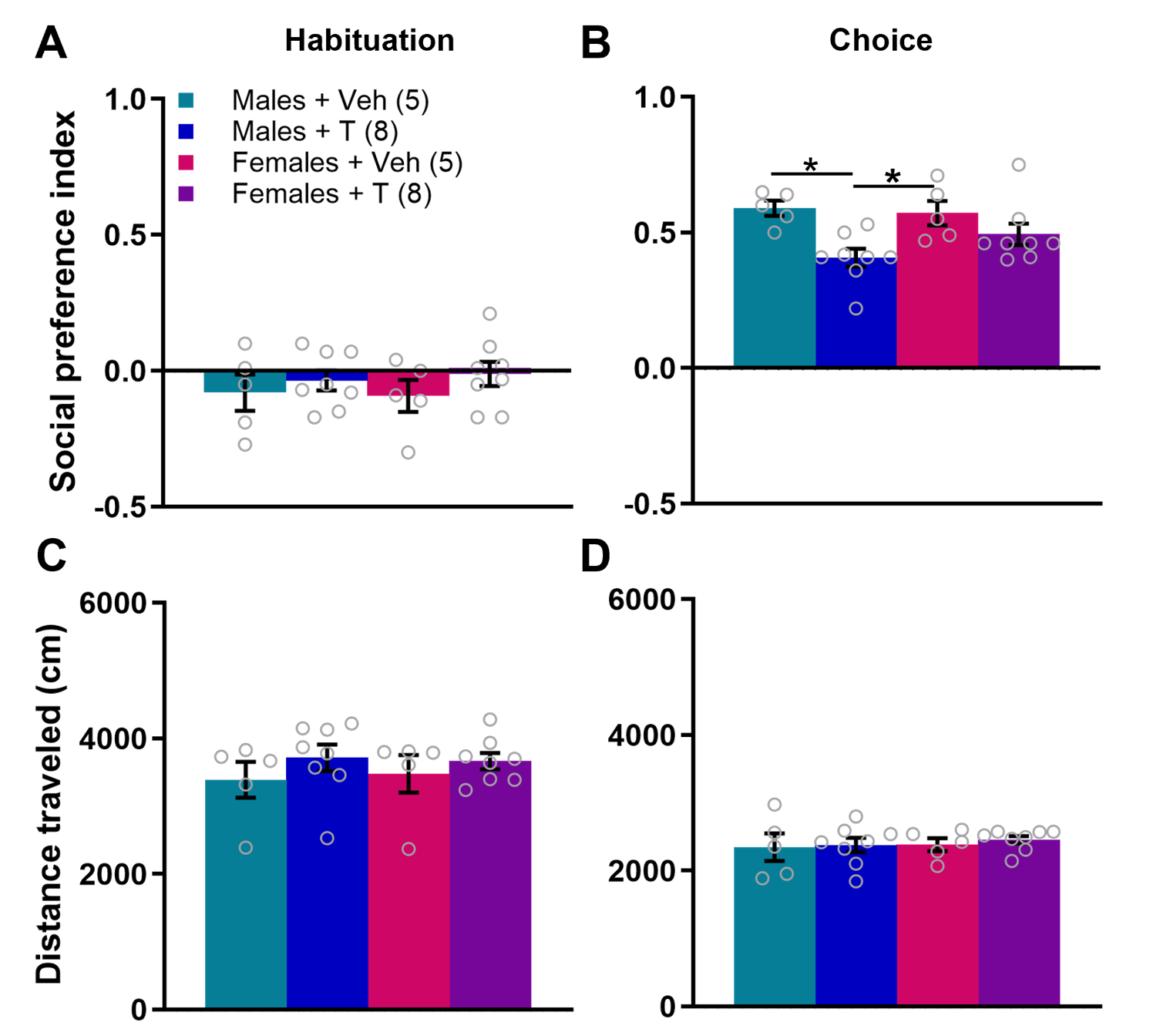

Supplement: Figure 2-1 — Testosterone administration on the day of birth induces social approach deficits in adolescent males. (A) During a 10 min habituation period of the social approach test, all experimental groups had a similar preference index (PI). A two-way ANOVA uncovered no significant main effect of sex (F(1, 22)=1.542, p=0.227, ƞ2 = 0.005) or treatment (F(1, 22) = 0.013, p = 0.909, ƞ2 = 0.0005), and no significant interaction (F(1, 22) = 0.125, p = 0.728, ƞ2 = 0.005). (B) Males treated neonatally with testosterone had a significantly lower social preference index than males or females treated with vehicle during the choice phase of the social approach test. A two-way ANOVA uncovered a significant main effect of sex (F(1, 22) = 11.130, p = 0.003, ƞ2 = 0.306) no main effect of treatment (F(1, 22) = 0.0762, p = 0.392, ƞ2 = 0.021), and no significant interaction (F(1, 22) = 1.779, p = 0.196, ƞ2 = 0.049). A Tukey post hoc test indicated that males treated on PN0 with testosterone (Males + T) had significantly lower freezing than Males + Veh and Females + Veh, p = 0.016 and 0.033, respectively. (C) There were no differences across groups in distance traveled during the social approach test in the habituation phase. A two-way ANOVA uncovered no significant main effect of sex (F(1, 22) = 0.009, p = 0.925, ƞ2 = 0.0003) or treatment (F(1, 22) = 1.521, p = 0.231, ƞ2 = 0.064), and no sex x treatment interaction (F(1, 22) = 0.107, p = 0.747, ƞ2 = 0.005). (D) There were no differences across groups in distance traveled during the social approach test in the choice phase. A two-way ANOVA uncovered no significant main effect of sex (F(1, 22) = 0.247, p = 0.624, ƞ2 = 0.011) or treatment (F(1, 22) = 0.217, p = 0.646, ƞ2 = 0.010), and no sex x treatment interaction (F(1, 22) = 0.028, p = 0.870, ƞ2 = 0.001). *p<0.05. Bars indicate mean ± SEM. Data points represent litter averages. Download Figure 2-1, TIF file. [file eneuro-12-ENEURO.0020-25.2025-s003.tif]

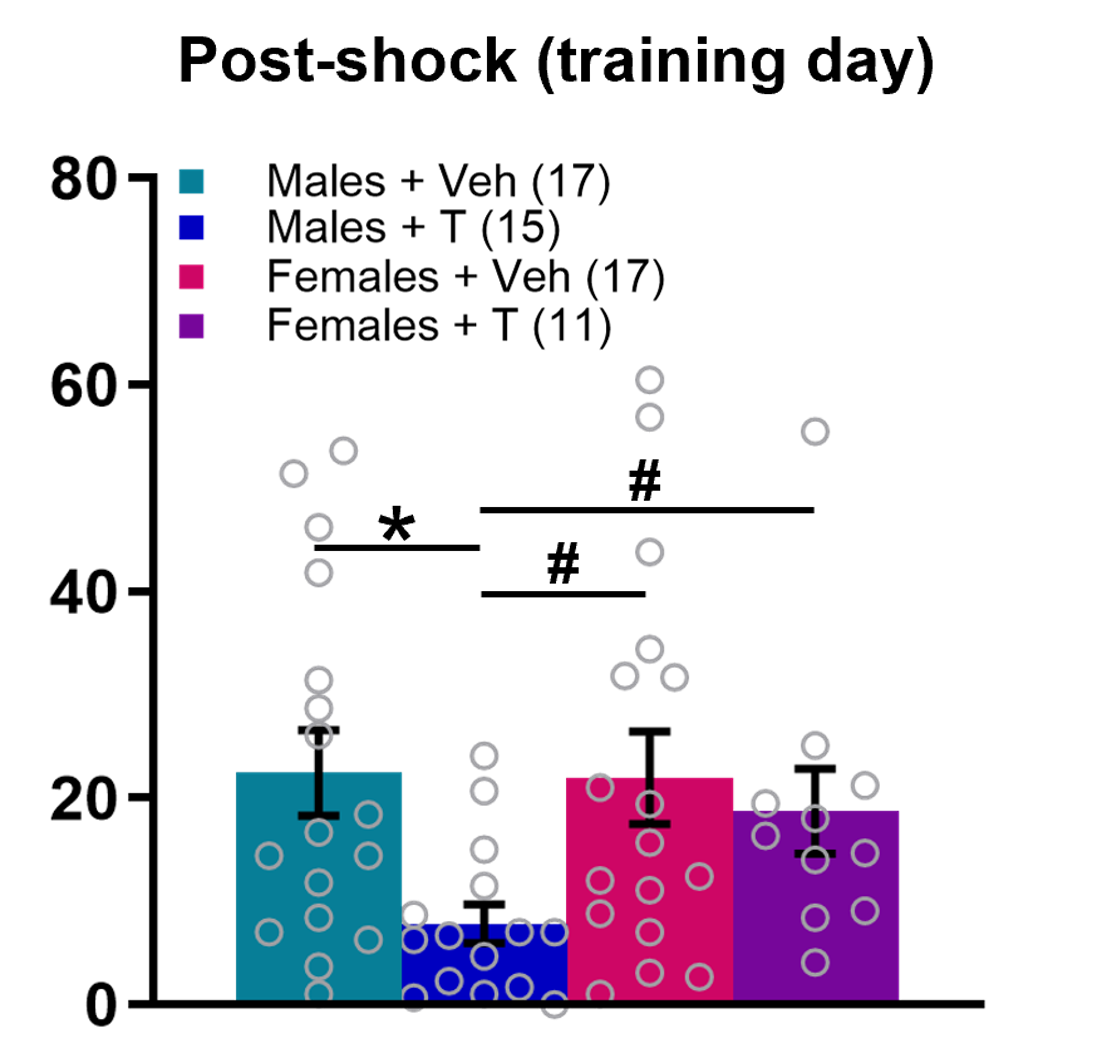

Supplement: Figure 3-1 — Adult males treated with testosterone on PN0 exhibited significantly less freezing during the 30 s following a single footshock on the training day of contextual fear conditioning. A two-way ANOVA uncovered trends towards significance for main effects of both sex (F(1, 55) = 3.034, p = 0.087, ƞ2 = 0.047) and treatment (F(1, 55) = 2.961, p = 0.091, ƞ2 = 0.045), and a significant interaction (F(1, 55) = 4.280, p = 0.043, ƞ2 = 0.066). A Tukey post hoc test indicated that males treated on PN0 with testosterone (Males + T) had significantly lower freezing than Males + Veh (p = 0.036) and a trend towards significantly less freezing than Females + Veh, p = 0.065 and Females + T, p = 0.071. *p<0.05, #p<0.10. Bars indicate mean ± SEM. Data points represent individual mice. Download Figure 3-1, TIF file. [file eneuro-12-ENEURO.0020-25.2025-s004.tif]

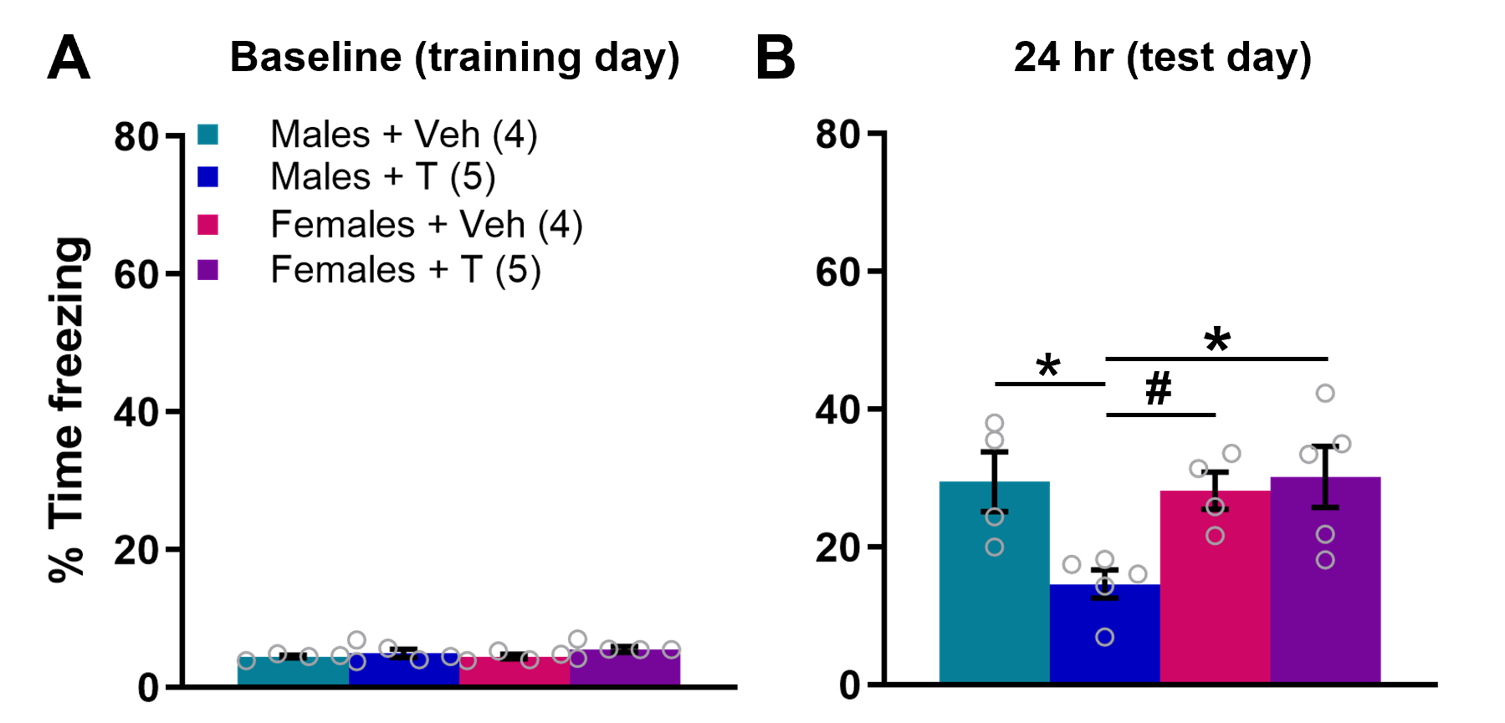

Supplement: Figure 3-2 — Testosterone administration on the day of birth induces fear memory deficits in adult males. (A) Baseline freezing prior to contextual fear conditioning was similar regardless of sex or treatment. A two-way ANOVA uncovered no significant main effect of sex (F(1, 14) = 0.449, p = 0.514, ƞ2 = 0.025) or treatment (F(1, 14) = 2.887, p = 0.111, ƞ2 = 0.162), and no sex x treatment interaction (F(1, 14) = 0.389, p = 0.543, ƞ2 = 0.022). (B) Adult males treated with T on PN0 exhibited significantly less freezing during the 24 hr memory test than those treated with veh or females treated with T. A two-way ANOVA uncovered a trend toward a main effect of sex (F(1, 14) = 3.985, p = 0.066, ƞ2 = 0.142) and treatment (F(1, 14) = 3.257, p = 0.093, ƞ2 = 0.116), and a significant interaction (F(1, 14) = 5.609, p = 0.033, ƞ2 = 0.200). A Tukey post hoc test indicated that Males + T had significantly lower freezing than Males + Veh (p = 0.046) and Females + T (p = 0.025), and a trend towards significantly less freezing than Females + Veh (p = 0.074). *p<0.05, #p<0.10. Bars indicate mean ± SEM. Data points represent litter averages. Download Figure 3-2, TIF file. [file eneuro-12-ENEURO.0020-25.2025-s005.tif]

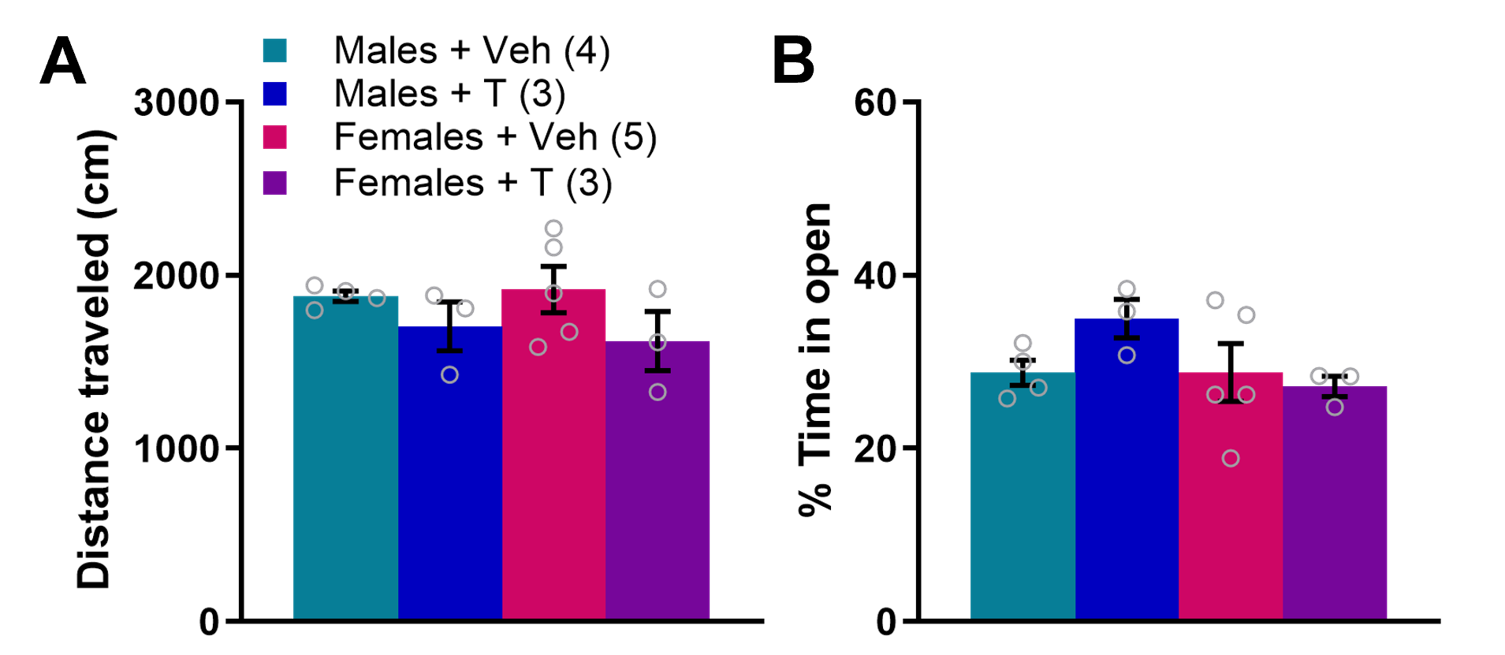

Supplement: Figure 4-1 — Neonatal testosterone treatment does not affect behavior in the elevated zero maze. (A) Naïve adult males and females treated neonatally with testosterone traveled similar distances in the elevated zero maze as those treated with veh. A two-way ANOVA uncovered a trend toward a main effect of sex (F(1, 11) = 3.297, p = 0.097, ƞ2 = 0.226) no significant effect of treatment (F(1, 11) = 0.032, p = 0.861, ƞ2 = 0.002), and no significant sex x treatment interaction (F(1, 11) = 0.232, p = 0.639, ƞ2 = 0.015). (B) Testosterone treatment on the day of birth did not affect percent time spent in the open arms of the EZM. A two-way ANOVA revealed no significant main effects (sex: F(1, 11) = 0.732, p = 0.411, ƞ2 = 0.048, treatment: F(1,11) = 2.073, p = 0.178, ƞ2 = 0.136), nor an interaction (F(1, 11) = 2.109, p = 0.174, ƞ2 = 0.138). Data points represent litter averages. Download Figure 4-1, TIF file. [file eneuro-12-ENEURO.0020-25.2025-s006.tif]

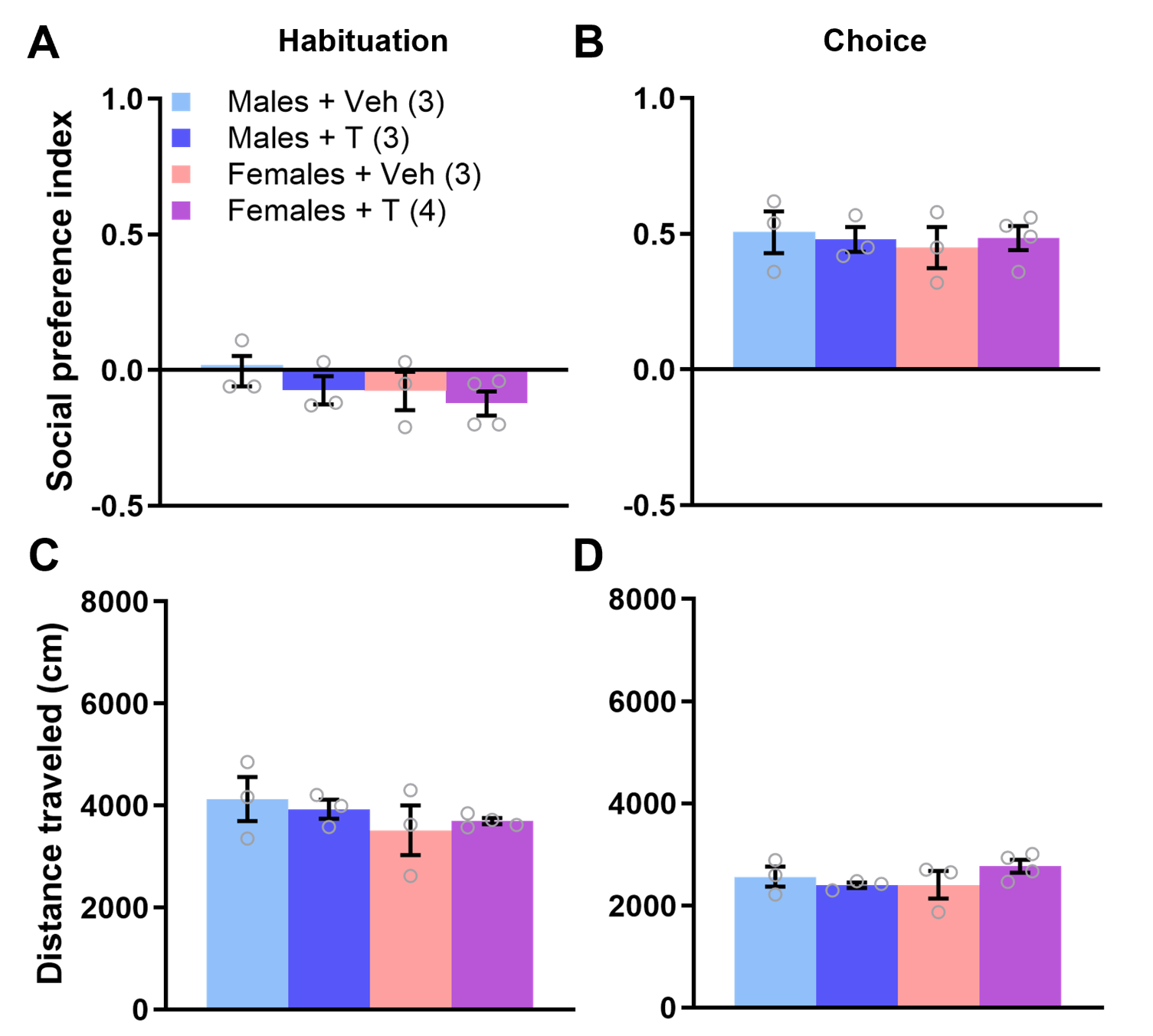

Supplement: Figure 5-1 — Testosterone administered on PN18 does not affect social approach behavior in juveniles. (A) Male and female adolescent mice exhibited no significant differences in PI during habituation regardless of treatment with veh or T on PN18. A two-way ANOVA revealed no significant main effects of sex (F(1, 9) = 1.210, p = 0.300, ƞ2 = 0.106) or treatment (F(1, 9) = 1.082, p = 0.325, ƞ2 = 0.094), and no significant interaction (F(1, 9) = 0.125, p = 0.728, ƞ2 = 0.005). (B) Testosterone treatment on PN18 had no effect on PI during the choice phase. A two-way ANOVA uncovered no significant main effects of sex (F(1, 9) = 0.183, p = 0.679, ƞ2 = 0.019) or treatment (F(1, 9) = 0.005, p = 0.947, ƞ2 = 0.0005), and no significant interaction (F(1, 9) = 0.260, p = 0.622, ƞ2 = 0.028). (C) During habituation, all experimental groups traveled similar distances. A two-way ANOVA uncovered no significant main effects of sex (F(1, 9) = 1.828, p = 0.209, ƞ2 = 0.165) or treatment (F(1, 9) = 0.001, p = 0.976, ƞ2 = 0.000), and no significant interaction (F(1, 9) = 0.361, p = 0.563, ƞ2 = 0.033). (D) During the choice phase, all experimental groups traveled similar distances. A two-way ANOVA revealed no significant main effects of sex (F(1, 9) = 0.378, p = 0.554, ƞ2 = 0.030) or treatment (F(1, 9) = 0.326, p = 0.582, ƞ2 = 0.026), and no significant interaction (F(1, 9) = 2.362, p = 0.159, ƞ2 = 0.190). Data points represent litter averages. Download Figure 5-1, TIF file. [file eneuro-12-ENEURO.0020-25.2025-s007.tif]

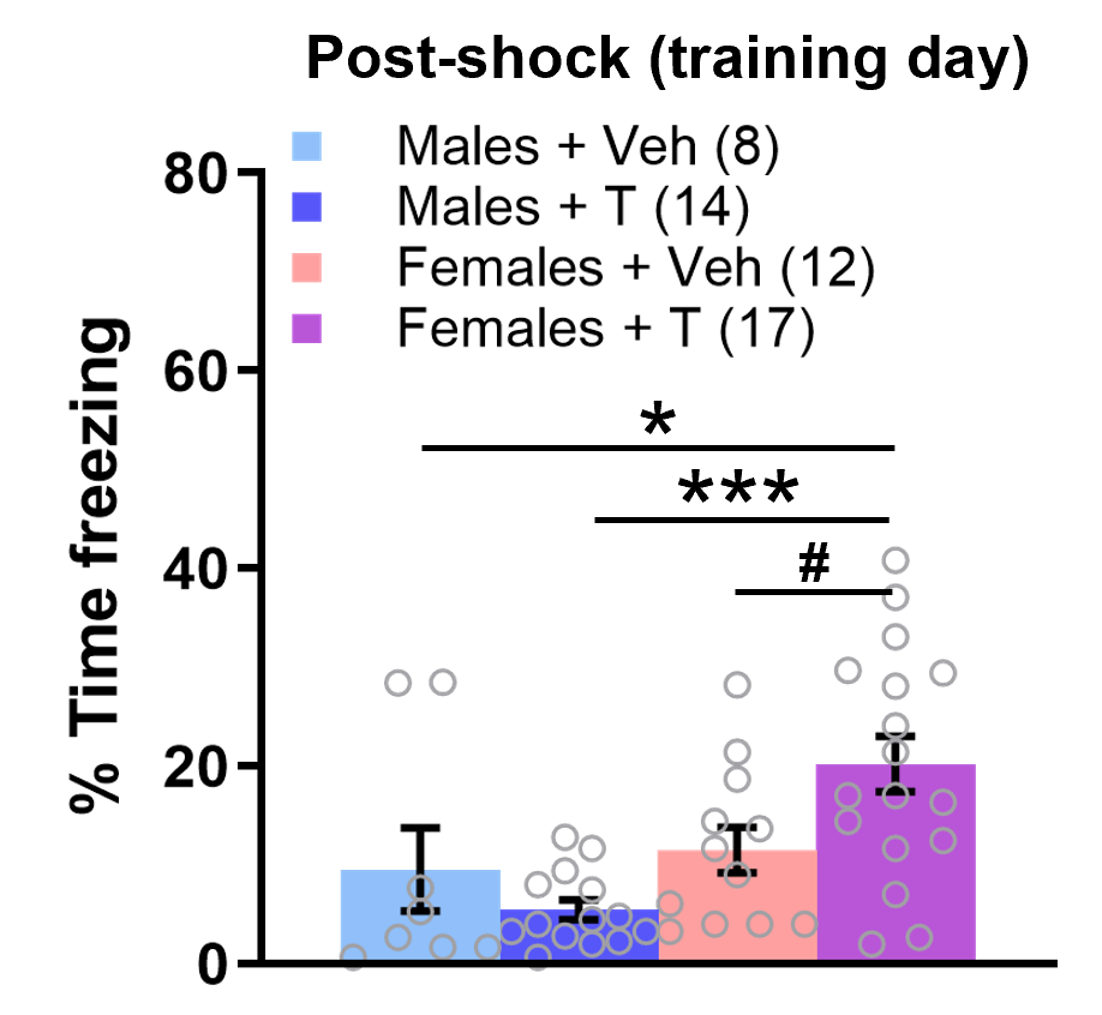

Supplement: Figure 6-1 — Testosterone administration on the day of birth causes increased freezing in females in the 30 s following a single footshock on the training day of contextual fear conditioning. A two-way ANOVA uncovered a significant main effect of sex (F(1, 47) = 9.629, p = 0.003, ƞ2 = 0.142) but not treatment (F(1, 47) = 0.742, p = 0.394, ƞ2 = 0.011), and a significant sex x treatment interaction (F(1, 47) = 5.651, p = 0.022, ƞ2 = 0.083). A Tukey post hoc test indicated that females treated on PN0 with testosterone had significantly higher freezing than Males + Veh (p = 0.047) and Males + T (p = 0.0003), and a trend towards significance compared to Females + Veh (p = 0.073). *p<0.05, ***p<0.001, #p<0.10. Bars indicate mean ± SEM. Data points represent individual mice. Download Figure 6-1, TIF file. [file eneuro-12-ENEURO.0020-25.2025-s008.tif]

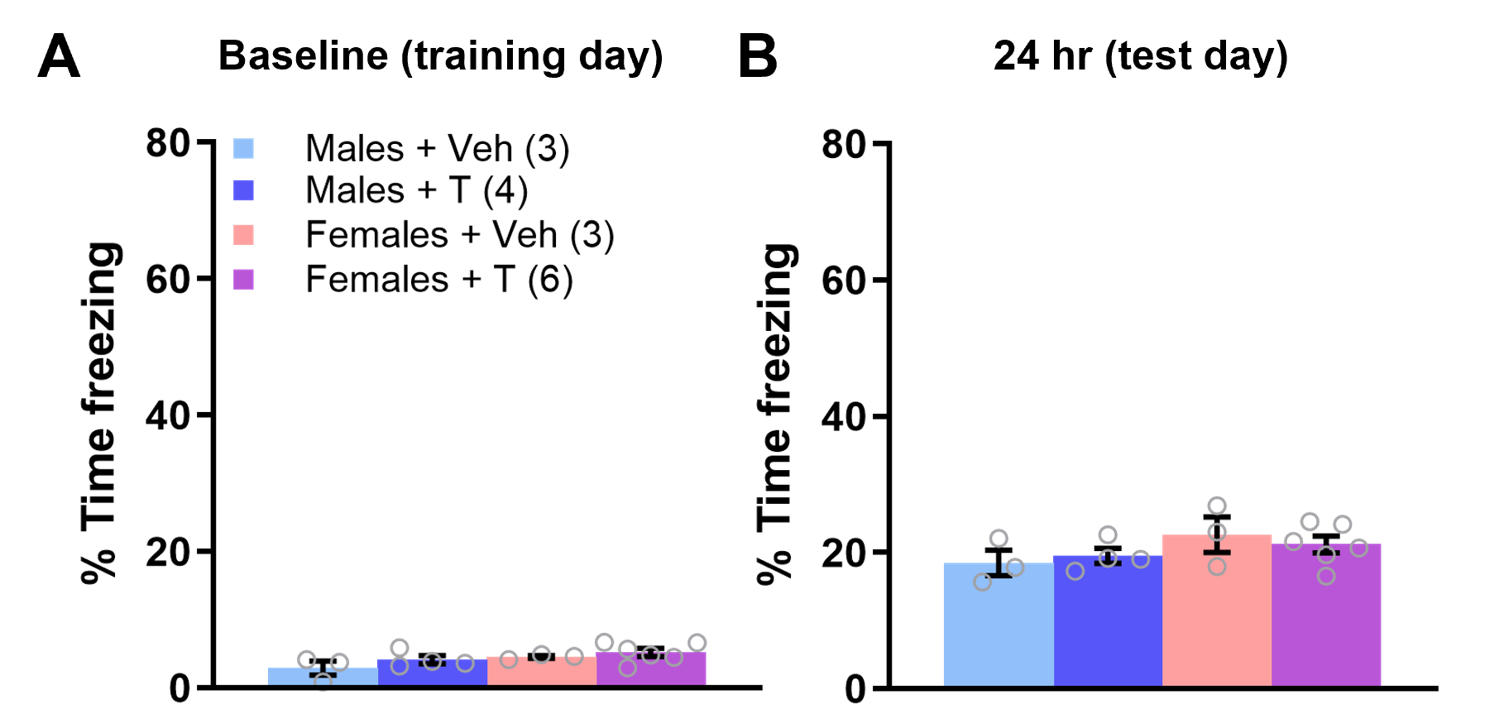

Supplement: Figure 6-2 — Testosterone administration on PN18 does not affect contextual fear conditioning in adults. (A) Treatment with T on PN18 did not affect percent of time spent freezing during the baseline measure. A two-way ANOVA uncovered a trend toward a main effect of sex (F(1, 12) = 3.725, p = 0.078, ƞ2 = 0.206), no main effect of treatment (F(1, 12) = 1.899, p = 0.193, ƞ2 = 0.105), and no significant interaction (F(1, 12) = 0.161, p = 0.695, ƞ2 = 0.009). (B) There were no group differences in freezing during the 24 hr test of contextual fear memory. A two-way ANOVA revealed no significance in main effects of sex (F(1, 12) = 3.071, p = 0.105, ƞ2 = 0.202) or treatment (F(1, 12) = 0.016, p = 0.901, ƞ2 = 0.001), and no sex x treatment interaction (F(1, 12) = 0.540, p = 0.477, ƞ2 = 0.036). Bars indicate mean ± SEM. Data points represent litter averages. Download Figure 6-2, TIF file. [file eneuro-12-ENEURO.0020-25.2025-s009.tif]
